# The Biosynthesis, Accumulation of Phenolic Compounds and Antioxidant Response in *Lactuca sativa* L. Plants Inoculated with a Biofertilizer Based on Soil Yeast and Iron Nanoparticles

**DOI:** 10.3390/plants13030388

**Published:** 2024-01-28

**Authors:** Daniela Berríos, Javiera Nahuelcura, Felipe González, Fabiola Peña, Pablo Cornejo, José Pérez-Navarro, Sergio Gómez-Alonso, Antonieta Ruiz

**Affiliations:** 1 Departamento de Ciencias Químicas y Recursos Naturales, Scientific and Technological Bioresource Nucleus BIOREN-UFRO, Universidad de La Frontera, Temuco 4811230, Chile; 2 Programa de Doctorado en Ciencias Agroalimentarias y Medioambiente, Facultad de Ciencias Agropecuarias y Forestales, Universidad de La Frontera, Temuco 4811230, Chile; 3 Programa de Doctorado en Ciencias Mención Biología Celular y Molecular Aplicada, Facultad de Ciencias Agropecuarias y Forestales, Universidad de La Frontera, Temuco 4811230, Chile; 4 Escuela de Agronomía, Facultad de Ciencias Agronómica y de los Alimentos, Pontificia Universidad Católica de Valparaíso, Quillota 2260000, Chile; 5 Centro Regional de Investigación e Innovación para la Sostenibilidad de la Agricultura y los Territorios Rurales, CERES, La Palma, Quillota 2260000, Chile; 6 Instituto Regional de Investigación Científica Aplicada, Universidad de Castilla-La Mancha, 13001 Ciudad Real, Spain

**Keywords:** phenolic compounds, antioxidant activity, lettuce, iron oxide nanoparticles

## Abstract

Lettuce is a vegetable that contributes vitamins, minerals, fibre, phenolic compounds and antioxidants to the human diet. In the search for improving production conditions and crop health, the use of microorganisms with plant growth-promoting capabilities, such as soil yeasts (PGPY), in conjunction with nanotechnology could offer sustainable development of agroecosystems. This study evaluated the synthesis of health-promoting bioactive compounds in lettuce under the application of soil yeast and an iron nanoparticle (NP-Fe_2_O_3_) encapsulated in alginate beads. Two yeast strains, *Candida guillermondii* and *Rhodotorula mucilaginosa*, and a consortium of both yeasts were used in the presence and absence of Fe_2_O_3_-NPs. Phenolic compounds were identified and quantified via HPLC-ESI-Q-ToF and antioxidant activity. Ten phenolic compounds were identified, highlighting the chicoric acid isomer and two quercetin glycosides with high concentrations of up to 100 µg g^−1^ in treatments with *C. guillermondii*. Treatments with *R. mucilaginosa* and NPs-Fe_2_O_3_ presented an increase in antioxidant activity, mainly in TEAC, CUPRAC and DPPH activities in leaves, with significant differences between treatments. Therefore, the use of encapsulated soil yeasts is a viable alternative for application in vegetables to improve the biosynthesis and accumulation of phenolic compounds in lettuce and other crops.

## 1. Introduction

Caloric intake via fruit and vegetable consumption has significantly changed in the last 10 years, increasing by 32% [1], and is considered a nutritious and essential food in the human diet [2,3], including the consumption of fermented vegetable food that also contribute to health benefits [4]. The benefits of vegetable consumption are directly related to the content of macro- and micronutrients and bioactive compounds such as polyphenols, flavonoids and carotenoids [5,6,7]. Lettuce (*Lactuca sativa* L.), belonging to the Asteraceae family, is one of the most important leafy vegetables, with 27.66 million tons produced worldwide in 2020 [8]. It is considered a healthy food because it is a good source of vitamins (B9, A, C, E and K), minerals, fibre [9], phenolic compounds and antioxidants [5,10]. Bioactive compounds present in foods such as lettuce are potentially associated with many beneficial health properties, such as anti-inflammatory, anti-diabetic, anticancer and even cardiovascular diseases [5,11].

There is evidence that phenolic compounds and antioxidants are involved in cellular defence against oxidative damage caused by free radicals [12]. It has been reported that the main phenolic compounds present in lettuce correspond to hydroxycinnamic acid, caffeic acid derivatives, caffeoylquinic acid and their isomers, chicoric acid and flavonols such as quercetin-3-glucuronide and quercetin–hexoside [10]. These compounds are synthesized normally during the plant’s growth cycle, but there is evidence that they can be induced when plants are subjected to certain abiotic stress conditions, such as drought or salt stress [10,13], and even light exposure conditions, such as shading [14]. In addition, Materska et al. (2019) [13] found that phenolic compounds play an important role in plant/plant and plant/pathogen signalling. Based on these conditions, plants generate a metabolic and biochemical response, represented as an increase in antioxidant enzymes, generating protection for plant tissues against oxidative damage [15].

Plants in general, including cropped plant species, have maintained a close association with soil microorganisms [16]. However, in the current scenario oriented to improve food production conditions in agriculture, rhizosphere microorganisms such as fungi, bacteria, actinomycetes and yeasts have been subjected to a wide characterization of their traits as plant growth promoter microorganisms (PGPMs) to be used as potential bioinoculants [17,18,19,20].

Currently, there is increasing interest in describing yeast strains with PGPY capabilities, considering the expression of traits such as the production of siderophores, organic acids, and enzymes such as 1-aminocyclopropane-1-carboxylic acid deaminase (ACC), as well as phosphate solubilization, translocation of macro- and micronutrients, improvement in photosynthetic activity and plant tolerance to abiotic stress [18,19,20,21,22]. New evidence suggests that yeast inoculation improves soil conditions, concomitantly increasing plant nutrient content and overall performance [20].

On the other hand, there are currently great advances in agriculture regarding the use of nanomaterials (NMs) [23] because nanoparticles (NPs) have small sizes, high surface/volume ratios, optical properties, low-cost formulations, and mainly high biological activity and controlled release kinetics at target sites [24,25]. This last property has allowed for the exploration and creation of nanoagrochemicals, nanopesticides and nanofertilizers, among others [26,27,28,29]. Specifically, nanofertilizers have acquired great relevance in the search for alternatives to replace the current use of conventional fertilizers, which require large amounts, as well as the use of water, to obtain higher yields and meet the demand for food [30]. Iron (Fe) is an essential nutrient for the growth and development of living organisms and plays an important role in biochemical and physiological processes [31]. Studies based on Fe NMs have shown that the controlled delivery of Fe generates a positive response in terms of seed germination, increased water uptake potential, improved biomass accumulation, photosynthetic processes, secondary metabolism and antioxidant enzyme activity [26,32,33] compared to traditional Fe-based fertilizers, thus improving physiological functions and even resistance to environmental stress [34,35,36].

Advances in agriculture via the incorporation of new NMs and microorganisms with PGP capabilities [37] highlight new and interesting alternatives focused on the encapsulation of NPs and microorganisms (biofertilizers), which are environmentally friendly [18,29]. Therefore, we hypothesized that the application of encapsulated NPs-Fe_2_O_3_ and soil yeasts could become an efficient strategy to improve the growth and metabolic behaviour of lettuce plants. In this sense, the objective of this work was to evaluate the effect on the biosynthesis of phenolic compounds and antioxidants in the lettuce crop under the application of encapsulated Fe NPs and different yeast strains with PGP capabilities.

## 2. Results

### 2.1. Determination and Quantification of Phenolic Compounds via HPLC-ESI-QToF in Lettuce Leaves

**Figure 1:**
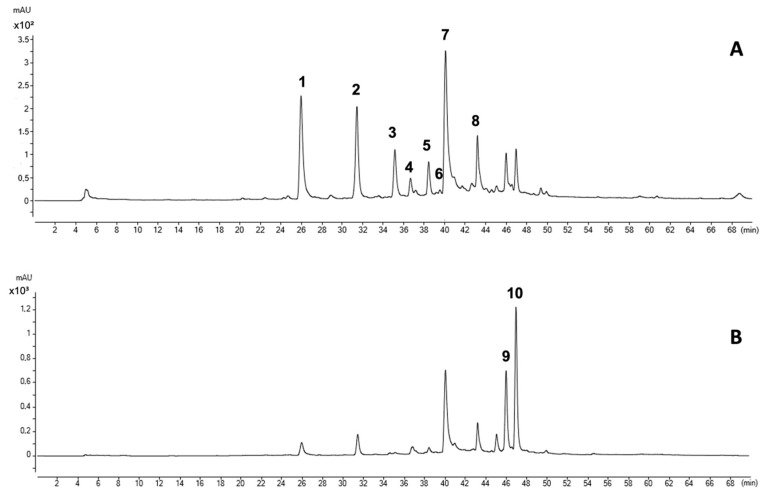
HPLC-ESI-Q-ToF chromatogram at 320 nm (**A**) and 360 nm (**B**) of phenolic compounds from lettuce leaves obtained from plants growing with the application of an encapsulation with soil yeasts and Fe₂O₃-NPs. The identifications are according to Table 1.

The phenolic compounds were identified via high-resolution mass spectrometry using an HPLC-ESI-Q-ToF system. Ten phenolic compounds were detected, corresponding to seven hydroxycinnamic acids, two flavonols and one unidentified compound (Table 1; Figure 1A,B). In the case of hydroxycinnamic compounds, two families were detected. The first corresponds to chlorogenic acid derivatives, where 5-caffeoylquinic acid (peak 2), caffeic acid derivate (peak 3), coumaroylquinic acid derivative (peak 4), caffeic acid derivate (peak 5) and chlorogenic acid derivative (peak 6) were identified based on their MS/MS spectra. Associated with the caftaric acid family, caftaric acid (peak 1) and the isomer of chicoric acid (peak 7) were identified based on their MS/MS spectra. Two compounds from the flavonol family were also identified as quercetin-3-glucoronide (peak 9) and quercetin acetyl hexoside derivative (peak 10) based on their MS/MS spectra. Additionally, peaks 1, 2 and 9 were identified via comparison with the retention times of their commercial standards.

The quantification of phenolic compounds was performed via external calibration with commercial standards of caftaric acid, chlorogenic acid and quercetin-3-glucoronide (Table 2). The highest concentrations of phenolic compounds were detected in the caftaric acid derivatives (Table 3). The isomer of chicoric acid (peak 7) presented the highest concentrations among all the hydroxycinnamic compounds, with an increase in T1 (*Candida* with NPs), T2 (*Candida* without NPs) and T3 (*Rhodotorula* with NPs) compared to the control without NPs (T0). Chlorogenic acids presented, in general, low concentrations, where 5-caffeoylquinic acid (peak 2) presented higher concentrations in the treatments T1 (53.64 µg g^−1^) and T2 (96. 80 µg g^−1^), both in the presence of the *Candida guillermondii* strain, and in T3 (*Rhodotorula* with NPs) and T6 (consortium without NPs), compared to the control without inoculum and NPs. Caffeic acid (peak 3) presented higher concentrations in treatments T1 and T3 than in the control without inoculum and NPs. Coumaroylquinic acid (peak 4) and the chlorogenic acid derivative (peak 6) presented the highest concentrations in T2 and T3 (Table 3). Regarding flavonols, quercetin 3-glucuronide (peak 9) and quercetin acetylhexoside derivative (peak 10) showed higher concentrations in the T2, T3 and T6 treatments than in the control, representing almost three to four times more flavonols than in T0 (Table 3).

### 2.2. Total Phenols and Antioxidant Activity in Leaves

**Figure 2:**
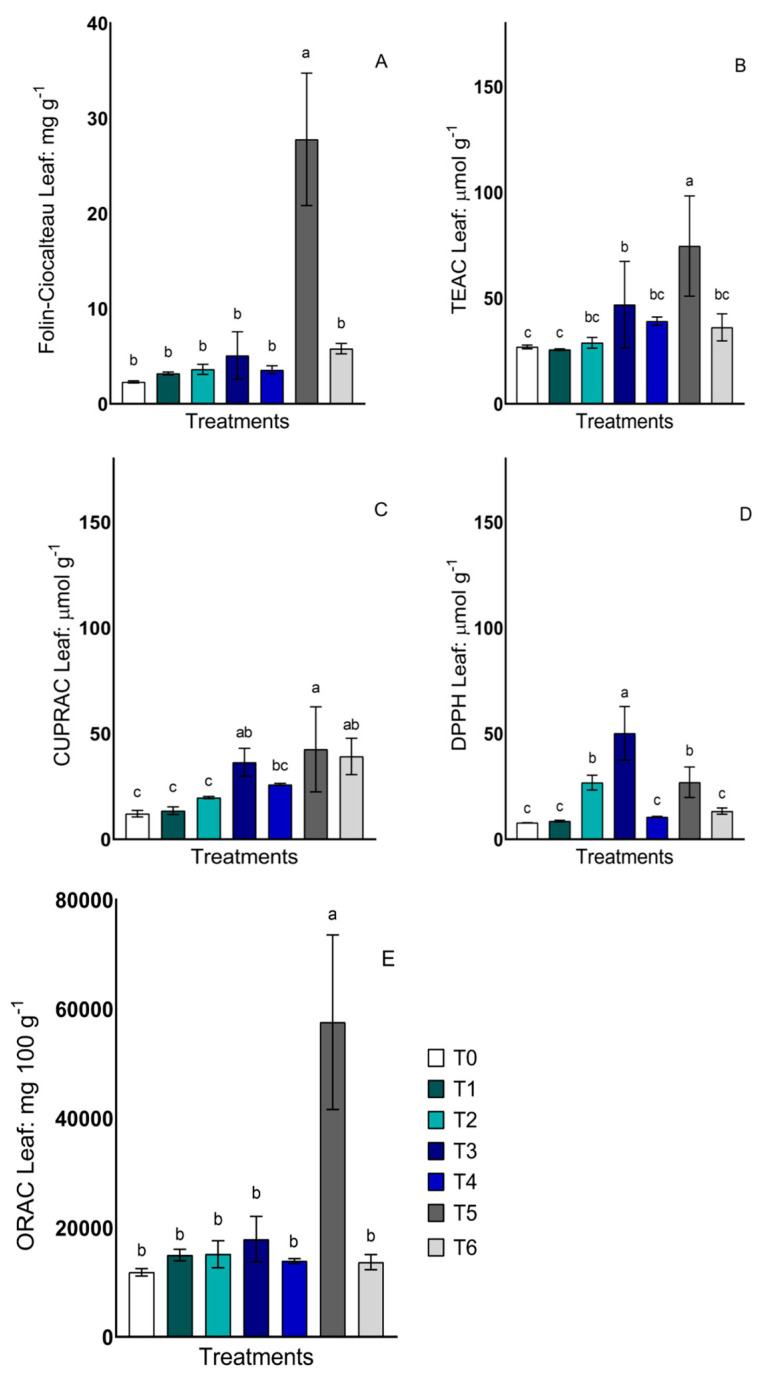
Total phenols and antioxidant activity in lettuce leaves of plants treated with two strains of yeast, *Candida guillermondii*, *Rhodotorula mucilaginosa* and a consortium, in the presence and absence of Fe nanoparticles. (**A**) Total phenols via the Folin–Ciocalteu method, (**B**) Trolox equivalent antioxidant capacity (TEAC), (**C**) cupric ion reducing antioxidant activity (CUPRAC), (**D**) antioxidant activity by 2,2-diphenyl-1-picrylhydrazyl (DPPH) free radical method, and (**E**) oxygen radical scavenging capacity (ORAC). The treatments described are as follows: Control without inoculum and NPs (T0); *Candida guillermondii* with NPs (T1); *Candida guillermondii* without NPs (T2); *Rhodotorula mucilaginosa* with NPs (T3); *Rhodotorula mucilaginosa* without NPs (T4); consortium with NPs (T5); consortium without NPs (T6). Values are means of four replicates ± standard errors (SE). Bars sharing the same lowercase letters between treatments are not significantly different from each other according to Tukey’s *p* < 0.05 post hoc test.

Total phenols and antioxidant activity were determined using the Folin–Ciocalteu, Trolox equivalent antioxidant capacity (TEAC), cupric ion reducing antioxidant activity (CUPRAC), antioxidant activity by the 2,2-diphenyl-1-picrylhydrazyl (DPPH) free radical method and oxygen radical scavenging capacity (ORAC) methods. In the determination of total phenols, only the treatment consortium with NPs (T5) presented an increase compared to the control (Figure 2A). Regarding the antioxidant activity determined via the TEAC method, a significant increase in antioxidant activity was also observed in T5, representing three times more than the control treatment (Figure 2B). CUPRAC presented a high activity in treatments T3, T5 and T6 compared to the control (Figure 2C). DPPH activity presented higher values in treatments T2, T3 and T5 (Figure 2D). The ORAC methodology responded similarly to Folin, with higher activity in the treatment T5 (Figure 2E).

### 2.3. Total Phenols and Antioxidant Activity in Roots

**Figure 3:**
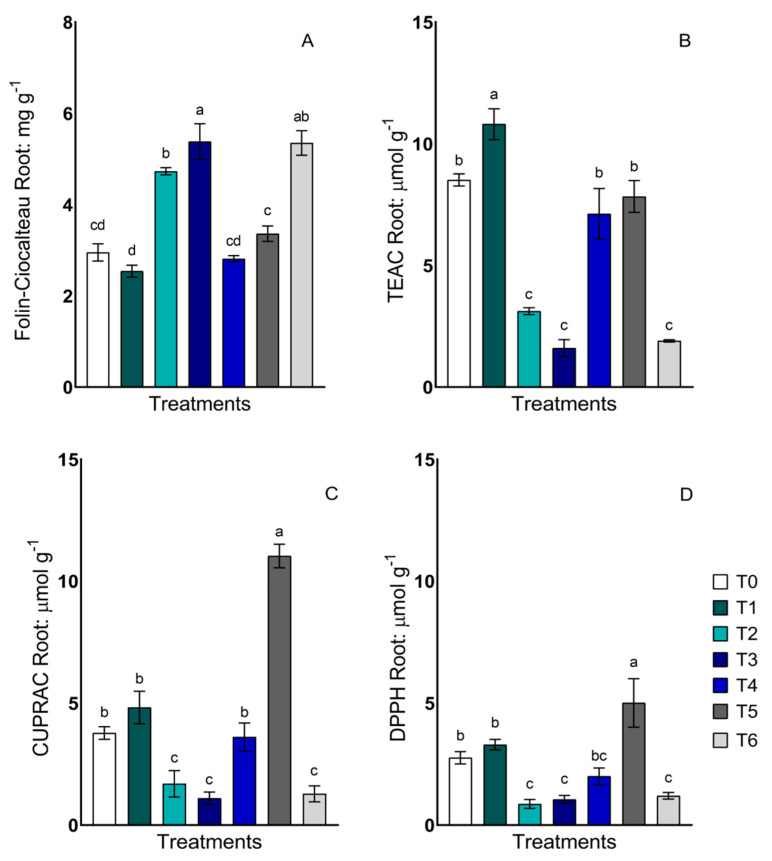
Total phenols and antioxidant activity in lettuce roots of plants treated with two strains of yeast, *Candida guillermondii*, *Rhodotorula mucilaginosa* and a consortium, in the presence and absence of Fe-nanoparticles. (**A**) Total phenols via Folin–Ciocalteu method, (**B**) Trolox equivalent antioxidant capacity (TEAC), (**C**) cupric ion reducing antioxidant activity (CUPRAC), (**D**) antioxidant activity by 2,2-diphenyl-1-picrylhydrazyl (DPPH) free radical method. The treatments described are as follows: control without inoculum (T0); *Candida guillermondii* with NPs (T1); *Candida guillermondii* without NPs (T2); *Rhodotorula mucilaginosa* with NPs (T3); *Rhodotorula mucilaginosa* without NPs (T4); consortium with NPs (T5); consortium without NPs (T6). Values are means of four replicates ± standard errors (SE). Bars sharing the same lowercase letters between treatments are not significantly different from each other, according to Tukey’s *p* < 0.05 post hoc test.

Total phenols and antioxidant activity were determined using the Folin–Ciocalteu, Trolox equivalent antioxidant capacity (TEAC), cupric ion reducing antioxidant activity (CUPRAC), antioxidant activity by the 2,2-diphenyl-1-picrylhydrazyl (DPPH) free radical method and oxygen radical scavenging capacity (ORAC) methods. The determinations of total phenols and antioxidant activities in roots were carried out using the same methodologies described for leaves, except for ORAC, which is an indicator preferentially used in food quality. Total phenolic compound concentrations responded differently between treatments. The highest concentrations were detected in treatments T2, T3 and T6, with important increases compared to the control T0 (Figure 3A). The antioxidant activity evaluated via the TEAC methodology (Figure 3B) presented significant differences among the treatments, where treatment T1 presented an activity 27% higher than T0, whereas in treatments T2, T3 and T6, decreases of 63.3%, 81.1% and 77.7%, respectively, were detected compared to the control. For CUPRAC (Figure 3C) and DPPH (Figure 3D) measurements, the treatment that responded with a higher antioxidant activity in the roots was treatment T5, with values of 11.02 µmol g^−1^ and 5.00 µmol g^−1^, respectively, representing a 192.3% and 81.3% increase compared to the control, respectively.

### 2.4. Multivariate Analysis

**Figure 4:**
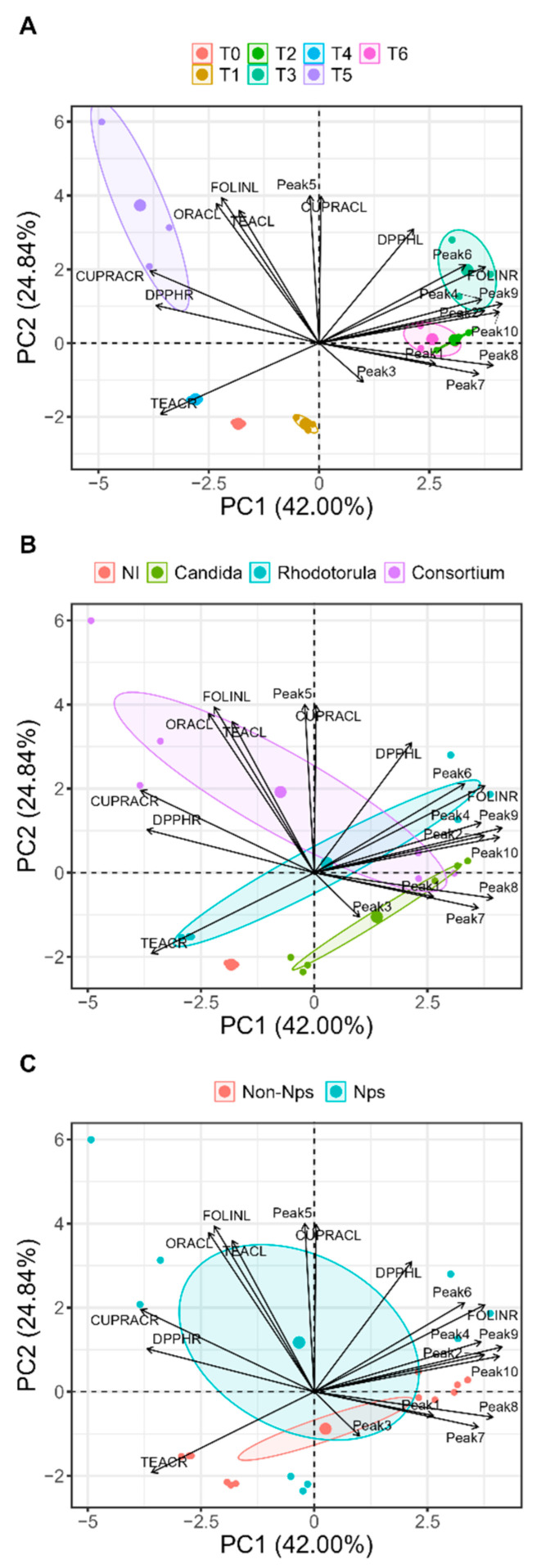
Principal Component Analysis (PCA) considering the overall behaviour of the different treatments (**A**), the evaluation of the different inocula used (**B**) the presence or absence of Fe nanoparticles (NPs) (**C**), where the treatments correspond to control without inoculum (T0); *Candida guillermondii* with NPs (T1); *Candida guillermondii* without NPs (T2); *Rhodotorula mucilaginosa* with NPs (T3); *Rhodotorula mucilaginosa* without NPs (T4); consortium with NPs (T5); consortium without NPs (T6). Phenolic compounds: peak 1: caftaric acid; peak 2: 5-caffeolquinic acid; peak 3: caffeic acid; peak 4: coumaroylquinic acid; peak 5: caffeic acid derivative; peak 6: chlorogenic acid derivative; peak 7: chicoric acid isomer; peak 8: unidentified; peak 9: quercetin-3-glucuronide; peak 10: quercetin acetylhexoside derivative and antioxidant activity in leaves and roots: Total phenols via Folin–Ciocalteu method, Trolox equivalent antioxidant capacity (TEAC), cupric ion reducing antioxidant activity (CUPRAC), antioxidant activity by 2,2-diphenyl-1-picrylhydrazyl (DPPH) free radical method and oxygen radical scavenging capacity (ORAC).

The factorial analysis using principal components yielded 66.8% of the total variance explained for PC1 (42%) and PC2 (24.8%). Figure 4A represents the behaviour of the treatments related to each of the experimental determinations. In the *Rhodotorula mucilaginosa* with NP treatment, clear associations were observed between the Folin values in roots and some phenolic compounds from the hydroxycinnamic acid family evaluated in leaves, such as the derivatives of chlorogenic acid, coumaroylquinic acid and 5-caffeolquinic acid. In Figure 4B, the associations of the different inocula applied and the different experimental variables were also evaluated. The treatments with the consortium are strongly associated with traits of antioxidant activity and total phenolic compounds in leaves, whereas the treatments with the inoculation of *C. guillermondii* are mainly associated with two phenolic compounds, caftaric acid and caffeic acid. The treatments containing *R. mucilaginosa* were clustered with characteristics evaluated in leaves, specifically phenolic compounds such as chlorogenic acid derivative and coumaroylquinic acid, antioxidant activity (TEAC) and total phenols in roots. Regarding the PC in Figure 4C, the variables associated with the presence and absence of nanoparticles are observed. The variable presence of nanoparticles is mainly associated with most of the evaluated parameters, such as TEAC in leaves and ORAC and CUPRAC in leaves. The absence of nanoparticles does not indicate interactions between the evaluated parameters.

## 3. Discussion

Recent studies have focused on the role of inoculants based on bacteria and fungi, but the potential of yeasts as potent plant growth promoters is poorly understood [38]. There are some studies related to the effects of yeast inoculation in crops, but they are mainly oriented towards evaluating PGPY capacities in maize, rice, black chickpea beans, grapes, pomegranate and tomatoes [39,40,41,42,43,44] via the determination of chlorophyll and carotenoid content and minerals, among other variables. Therefore, as far as we know, this is the first work that evaluates bioactive compounds in lettuce inoculated with soil yeast and iron nanoparticles.

The metabolic profile in lettuce will vary depending on some factors, such as cultivar, leaf colour, whether green or red/green, and whether the leaves are subjected to some type of stress that can change the production of polyphenols, flavonols, anthocyanins, and antioxidant capacity, among others [10,45,46]. In the present study, carried out in romaine-type lettuce, ten phenolic compounds were identified, most of them corresponding to hydroxycinnamic acids associated with two families, the first to caftaric acid and the second to chlorogenic acid. The other group corresponds to flavonols, specifically quercetins, compounds that were previously reported in different studies performed in five cultivars of lettuce and endive growing under field conditions [47], in normal- and baby-size lettuce growing under greenhouse conditions [48], in lettuce plants subjected to salinity stress [21] and in different cultivars and climatic seasons of red lettuce growing under greenhouse conditions [49].

The isomer of chicoric acid was the compound quantified with higher concentrations, mainly in treatments T1 and T2, both treatments inoculated with the yeast *C. guillermondii*; moreover, the results reported here are higher than those obtained by Santander et al. (2020) [21] using lettuce belonging to cultivar Lollo Bionda and inoculated with *Claroideoglomus claroideum*, reaching concentrations of 120 µg g^−1^, and higher than those reported by Fincheira et al., (2023) [50], which showed concentrations of 2–9 µg g^−1^ of chicoric acid in lettuce plants supplied with lipid nanoparticles as a carrier for two ketones. In addition, Gonzalez et al. (2023) [51] inoculated lettuce plants with *Actinobacteria* spp. obtained high levels of phenolic compounds, reaching values of up to 150 mg g^−1^ of chicoric acid in plants subjected to salt stress and between 35 and 75 mg g^−1^ in plants without stress.

Flavonols also presented high concentrations, specifically quercetin-3-glucoronide, in the treatments inoculated with *C. guillermondii* and with *R. mucilaginosa*, also showing concentrations higher than those reported by Santander et al. (2020 and 2022) [10,21] in different lettuce cultivars inoculated with two different arbuscular mycorrhizal fungi and subjected to salt stress (12 and 20 µg g^−1^). This could be explained because the production of phenolic compounds is modulated by factors such as plant genotype, the presence of PGPM, and biotic or abiotic stress factors, including the presence of metallic nanoparticles [10,50,51,52].

On the other hand, the Folin–Ciocalteu method is considered a nonspecific procedure for phenolic compounds because it can respond to organic compounds of different natures, such as sugars, proteins or ascorbic acid, in addition to other inorganic substances, such as Fe^2+^ ions [53]. This behaviour can explain the response observed in most of the treatments. Santander et al. (2020) [21] also reported concentrations of approximately 1 mg g^−1^ gallic acid equivalents (GAE), similar to the concentrations reported here in most of the treatments, except for the treatment Consortium with NPs (T5), which generally presented low concentrations of hydroxycinnamic acids and flavonols.

Different procedures to report antioxidant capacities are commonly used to evaluate the response of the plant defence system [54]. Specifically, the 2,2-diphenyl-1-picrylhydrazyl radical (DPPH), Trolox equivalent antioxidant activity (TEAC) and cupric ion reducing antioxidant activity (CUPRAC) were evaluated in leaves and roots to provide a complete visual regarding the elimination of free radicals [50]. The oxygen radical absorbance capacity (ORAC) was evaluated only in leaves. In leaves, the TEAC and CUPRAC assays showed that the treatments inoculated with *R. mucilaginosa* and the consortium with Fe NPs (T3 and T5) displayed a marked response as an antioxidant defence mechanism. It is also important to note that there is a correlation between TEAC and CUPRAC with the content of phenolic compounds, suggesting that this type of compound is mainly responsible for antioxidant activity [55]. Regarding the above, the treatments *C. guillermondii* with NPs (T2) and *R. mucilaginosa* with NPs (T3) showed high free radical scavenging capacity. However, there are no related studies that have evaluated the antioxidant capacities of both strains. There are some other studies using other types of microorganisms. For instance, Santander et al. (2022) [10] observed an increase in DPPH activity in lettuce subjected to salt stress and inoculated with different AMF, where a close relationship between the increase in DPPH activity, phenolic compounds and concentrations of flavonoids was reported, similar to those obtained in this study. Regarding the antioxidant activity of ORAC, there were no differences between the treatments, except for the T5 treatment. Despite this, the values obtained here were higher than those reported by Avio et al. (2017) [56] in different lettuce cultivars inoculated with AMF and at two harvest times, which reported values in the range of 1.4–1.8 µmol TE 100 g^−1^, very low compared to this work.

The determination of total phenols in roots showed that treatments with *C. guillermondii* without NPs (T2), *R. mucilaginosa* with NPs (T3) and consortia without NPs (T6) presented higher concentrations of total phenolic compounds compared to the control, with treatment T3 presenting the highest concentration (5.38 mg GA g^−1^). This result could be explained in two ways: (i) *R. mucilaginosa* has been widely described as a strain with several PGP traits [18], and (ii) diverse studies have indicated that metallic oxide-type NPs produce increases in total phenol concentrations [57]. Therefore, when plants are exposed to NPs, an increase in the production of antioxidant compounds, such as phenolic compounds, is observed [58]. In addition, studies performed with *Saccharomyces cerevisiae* yeast strains evaluating their growth inhibition via oxygenation and under the application of different nanoparticles, such as TiO_2_, Fe_2_O_3_, SiO_2_, Al_2_O_3_ and CeO_2_, showed zero inhibition activity of O_2_ and membrane damage [59,60]. On the other hand, there are other NPs that can inhibit *S. cerevisiae*; therefore, the capacity of the yeast strain and the type of NPs supplied can determine the accumulation of phenolic compounds and total phenols [10,58,61].

At present, there is no clear information about the mechanisms that could better explain the interactions that may occur between nanoparticles or yeast and plants. Recently, Perez et al. (2023) [62] reported that the effects of soil yeasts and other PGP microorganisms are similar to those reported in this study regarding PGPY application on phenolic compounds mainly, where PGP yeasts significantly affect non-enzymatic antioxidant responses, decreasing total phenol concentrations, but increasing flavonol concentrations. In our work, similar results were obtained where flavonols such as quercetins had high concentrations compared to hydroxycinnamic acids; therefore, it could be suggested that there is a possible alteration in the biosynthesis of phenolic compounds induced by yeast inducing the biosynthesis of flavonols.

The use of biofertilizers is a sustainable alternative to traditional fertilizers that affect living organisms and the environment [63]. The use of these new types of biofertilizers, based on bacteria, fungi or yeasts, positively improve parameters such as germination, growth, yield and quality of crops [64]. Although the use of soil yeasts is based on their PGPY capabilities, promising results have been discussed in the improvement in agricultural practices, mainly in the decrease in the use of fertilizer use and also in the way of application of these new technologies such as nanofertilizers, mainly because they have proven to be effective supplying nutrients to crops in a controlled way [65]. This type of biotechnological tool is even more interesting if it works in conjunction with soil yeasts, highlighting their importance as more suitable microbial candidates as PGP since they are not only able to increase plant growth, provide protection against plant pathogens and reduce abiotic stress, but are also recognized as safe for use in agriculture (GRAS) [38,66]. These new, more sustainable alternatives offer a possibility of better food production, especially vegetables in which an increase in consumption has been evidenced [67], where lettuce plants are vegetables with high nutritional value and low caloric value and contain a wide range of bioactive compounds such as phenolic compounds such as hydroxycinnamic acids and flavonols, which are vital for health [13].

Finally, as this study is one of the first to demonstrate beneficial effects by using different yeasts as bioinoculants, mainly at the biochemical level, new approaches, including other plant responses, must be performed. Additionally, the use of yeast as a component in microbial consortia together with other complementary technologies, such as nanomaterials, can be a good starting point to develop more efficient biotechnological and sustainable tools not only oriented to increase plant growth but also to enhance the mechanisms that provide tolerance to globally increasing environmental stresses.

## 4. Materials and Methods

### 4.1. Experimental Design and Growing Conditions

A full randomized factorial design was used, including seven treatments and six replicates (N = 42). The yeast species used for encapsulation were *Candida guillermondii* and *Rhodotorula mucilaginosa*.

Both strains were collected from soil coming from mining tailings from the Piuquenes nonoperational reservoir, located in the Aconcagua Valley, Los Andes, Valparaíso Region (32°59′47.96″ S; 70°15′14.16″ W) near the Blanco River. The isolation and identification of both yeast strains was carried out according to Perez et al. (2023) [62]. Yeast strains were purified and resuspended in Yeast Extract Peptone Dextrose (YPD) medium, supplemented with 25% *v*/*v* glycerol and stored at −80 °C. Then, 100 μL of new YPD culture medium was used, and 100 μL of yeast was added according to treatment and incubated for 24 h at 28 ± 2 °C. Subsequently, the optical density (OD) was adjusted at 600 nm. The medium containing the yeasts was centrifuged for 10 min at 5000× *g*, and these were lyophilized before encapsulation. A mixture of 100 mL of whole milk/distilled water 70:30 was prepared and sterilized at 121 °C for 25 min, which was used as a lyoprotectant. Overall, 6 mL of the milk/water mixture was added to the pellet and vortexed. A stock solution of 1000 mg mL^−1^ of iron oxide/nanodust nanoparticles (α-Fe₂O₃, 99%) (SkySpring Nanomaterials, Inc. Houston, TX, USA) was prepared in 100 mL of deionized water and then an ultrasound of 130 watts at 70% amplitude for 5 min. 3% sodium alginate was used, which was sterilized at 121 °C for 21 min. Then, 100 mg of lyophilized yeast and nanoparticles were added to each treatment using a peristaltic pump FPP-Lab V3 (Biobase Group, Jinan, China) at a flow rate of 1 mL min^−1^. Finally, they were deposited in a calcium chloride solution where the capsules were formed in agitation and stored at 4 °C.

Each yeast and the consortium of both were encapsulated with and without nanoparticles (Fe₂O₃) depending on treatment, and an unencapsulated control treatment without yeast and without nanoparticles was used. The treatments used were as follows: T0, control without inoculum; T1, *C. guillermondii* with NPs; T2, *C. guillermondii* without NPs; T3, *R. mucilaginosa* with NPs; T4, *R. mucilaginosa* without NPs; T5, consortium with NPs; and T6, consortium without NPs. Romaine lettuce (cv. Bionda Degli Ortalani) green leaves were used as hosts, which were developed under controlled greenhouse conditions of a 16:8 light/dark photocycle, 18/26 °C night/day and 50/60% relative humidity. The plants were transplanted three weeks after germination into individual 0.5 L pots. The total period of the study was 52 days.

### 4.2. Identification and Quantification of Phenolic Compounds and Hydroxycinnamic Acids (HCAD) in Leaves by Using HPLC-ESI-QToF

The extraction procedure was carried out according to Llorach et al. (2008) [47] with some modifications. Briefly, leaf tissues were pulverized in carbon dioxide. One hundred milligrams of each tissue sample were mixed with 1.5 mL of extraction solvent (MeOH:water:formic acid 50:48.5:1.5 *v*:*v*:*v*) followed by 3 min in a sonication bath at 25 °C, shaken at 450 rpm for 1 h and centrifuged at 14,000 rpm for 5 min. The process was repeated two times. One millilitre of the total extraction was dried in a rotoevaporator, concentrated to 200 µL and stored at −20 °C until injection. The analytical system consisted of a 1260 Infinity high-performance liquid chromatography system coupled to a diode array detector (HPLC-DAD) (Agilent, Waldbronn, Germany) and a 6545-quadrupole time-of-flight (Q-ToF) mass spectrometer (Agilent, Waldbronn, Germany). The control software used was a Mass Hunter workstation (version B.06.11, Agilent Technologies, Inc., Santa Clara, CA, USA). A dual-jet electrospray ionization source (AJS-ESI dual) operated in negative ionization mode was used for Q-ToF. The methodology was carried out according to Favre et al. (2018) [68], where the parameters used were capillary voltage, 3500 V; gas temperature, 350 °C; drying gas, 8 L min^−1^; nebulizer, 40 psig; enveloping gas temperature, 400 °C; enveloping gas flow, 12 L min^−1^; acquisition range, 100–1000 *m*/*z*; and CID, linear range of 30–45. A total of 5 μL of each sample was injected onto an Ascentis Express C_18_ column (150 mm × 4.6 mm, 2.7 μm; Supelco Analytical, 595 North Harrison Road, Bellefonte, PA 16823, USA) at 16 °C. The solvents used were 0.1% HCOOH in water (solvent A) and 0.1% HCOOH in methanol (solvent B). The solvent A gradient was as follows: 2 min, 93%; 25 min, 68%; 40 min, 43%; 50 min 33%; 55 min, 3%; 65 min 3%; 70 min, 93%; with a flow rate of 0.300 mL min^−1^. The quantification of phenolic compounds was performed via external calibration at the maximum wavelength of each family of compounds (320 nm for hydroxycinnamic acids and 360 nm for flavonols) using caftaric acid, chlorogenic acid and quercetin-3-glucoronide as standards.

### 4.3. Total Phenols and Antioxidant Activity in Leaves and Roots

Determinations were performed according to Llorach et al. (2008) [47], with some modifications. Briefly, leaves and root samples were lyophilized and pulverized in liquid nitrogen, and 35 mg of each tissue was mixed with 1.5 mL of extraction solvent (MeOH:water:formic acid 25:24:3 *v*:*v*:*v*) followed by 1 min of ultrasound at 80% amplitude, shaken at 200 rpm for 20 min and centrifuged at 4000× *g* for 20 min. All spectrophotometric measurements were carried out in microplates using UV–visible Epoch equipment (BioteK, Winooski, VT, USA). The determination of total phenols was carried out using the Folin–Ciocalteu method. However, antioxidant activities were evaluated via Trolox equivalent antioxidant capacity (TEAC), cupric ion reducing antioxidant activity (CUPRAC), and antioxidant activity by 2,2-diphenyl-1-picrylhydrazyl (DPPH) free radical and oxygen radical scavenging capacity (ORAC) methods [10,69].

### 4.4. Statistical Analysis

All statistical analyses were conducted in R version 4.2.1. After verifying the normality and homoscedasticity of the data, the datasets were subjected to a two-way analysis of variance (ANOVA) with yeast inoculation and the presence of nanoparticles as sources of variation. For variables showing significant differences, means were compared using Tukey’s HSD multiple range test, with a significance level of *p* < 0.05 established for all cases, employing the R library “agricolae” v.1.3.5. The means ± standard error was represented as bar charts, and statistical differences were denoted by different lowercase letters between treatments. Additionally, the dataset underwent principal component analysis (PCA). Confidence ellipses (group means) inoculation was generated using the “FactoMineR” v.2.7 and “factoextra” v.1.0.7 packages.

## 5. Conclusions

In this study, it was demonstrated that the use of an encapsulate containing yeast and nanoparticles favours the synthesis of antioxidant compounds in lettuce plants. Differences were observed between the control treatment and the treatments containing the *Candida guillermondii* strain, which showed a tendency to increase the levels of most of the phenolic compounds, especially chicoric acid and quercetin derivatives. Therefore, it can be suggested that this yeast strain has the potential to be used in bioinoculant formulations. The study of PGP yeasts, encapsulated together with Fe nanoparticles, as bioinoculants and their use as an alternative to synthetic fertilizers in agriculture is still under study, but our results highlight the possibility of using this type of microorganism as a real, sustainable alternative to be applied to different food crops to improve productivity and concomitantly cope with climate change effects.

## Figures and Tables

**Table 1:** Identification of phenolic compounds in *Lactuca sativa* leaves via HPLC-ESI-ToF. Identifications according to Figure 1 and Figure 2.

Peak	Rt (min)	Identification	Λmax (nm)	[M − H]^−^	Productions
1	26.1	Caftaric acid	330	311	135.0, 149.0, 179.0, 112.0
2	31.5	5-Caffeoylquinic acid	325	295	191.0, 163.0
3	35.3	Caffeic acid derivate	330	135	135.0, 112.0
4	36.8	Coumaroylquinic acid derivative	325	337	-
5	38.4	Caffeic acid derivate	330	591	133.0, 179.0, 295.0
6	39.5	Chlorogenic acid derivative	332	337	163.0, 191.0, 206.0
7	40.4	Chicoric acid isomer	334	473	135.0, 149.0, 179.0, 293.0, 311.0
8	43.3	No identified	-	-	-
9	46.1	Quercetin 3-glucuronide	359	477	301.0
10	47.2	Quercetin acetyl hexoside derivative	360	505	301.0

**Table 2:** Analytical parameters in leaves and roots for chromatographic and spectrophotometric methods.

	Method	Standard	Equation	R^2^	DL	QL	LR	CV %
	Folin	Gallic acid	Y = 0.0007 x + 0.0332	0.9964	3.747 µM	12.491 µM	12.491–500 µM	87.65
	TEAC	Trolox	Y = 0.4157 x + 0.0183	0.9980	0.008 µM	0.027 µM	0.027–0.7 µM	48.67
Leaves	CUPRAC	Trolox	Y = 3.2264 x + 0.1414	0.9975	0.031 µM	0.105 µM	0.105–0.7 µM	98.30
	DPPH	Trolox	Y = 0.5678 x − 0.0034	0.9905	0.023 µM	0.076 µM	0.076–0.7 µM	81.24
	ORAC	Trolox	Y = 0.4155 x + 6.2634	0.9922	8.42 µM	28.06 µM	28–80 µM	87.07
	HPLC-ESI-QToF	Caftaric acid	Y = 1394.1 x − 245.61	0.9995	0.57 mg L^−1^	1.91 mg L^−1^	1.9–20 mg L^−1^	61.18
	HPLC-ESI-QToF	Chlorogenic acid	Y = 1738.2 x − 438.63	0.9991	0.75 mg L^−1^	2.53 mg L^−1^	2.5–20 mg L^−1^	60.59
	HPLC-ESI-QToF	Quercetin-3-glucoronide	Y = 1104.5 x −148.01	0.9981	0.76 mg L^−1^	2.52 mg L^−1^	2.5–20 mg L^−1^	70.71
	Folin	Trolox	Y = 0.0008 x + 0.0158	0.9963	7.349 µM	24.49 µM	24.49–500 µM	31.40
	TEAC	Trolox	Y = 0.4149 x + 0.0371	0.9933	0.020 µM	0.067 µM	0.067–0.7 µM	60.21
Roots	CUPRAC	Trolox	Y = 2.6455 x + 0.1776	0.9892	0.014 µM	0.048 µM	0.048–0.7 µM	85.74
	DPPH	Trolox	Y = 0.5761 x + 0.0138	0.9972	0.016 µM	0.055 µM	0.055–0.7 µM	67.13

Antioxidant methodologies evaluated: Total phenols using the Folin–Ciocalteu method and antioxidant activities, Trolox equivalent antioxidant capacity (TEAC), cupric ion reducing antioxidant activity (CUPRAC), antioxidant activity using the 2,2-diphenyl-free radical method. 1-picrylhydrazyl DPPH, oxygen radical scavenging capacity (ORAC). DL, detection limit; QL, quantification limit; LR, linear range; CV%, coefficient of variation.

**Table 3:** Individual phenolic compounds in leaves concentration (μg g^−1^) via HPLC-DAD.

Treatments	Peak 1	Peak 2	Peak 3	Peak 4	Peak 5	Peak 6	Peak 7	Peak 8	Peak 9	Peak 10
T0	25.98 ± 2.69 b	22.61 ± 3.73 d	6.85 ± 1.83 cd	7.78 ± 0.53 cd	6.93 ± 0.42 c	3.86 ± 0.10 cd	31.45 ± 15.64 c	10.95 ± 0.36 b	24.95 ± 0.48 de	27.63 ± 2.87 cd
T1	58.45 ± 6.73 a	53.64 ± 3.15 bc	48.82 ± 8.02 a	9.51 ± 0.41 bcd	18.18 ± 0.63 bc	4.12 ± 0.90 cd	135.13 ± 23.20 ab	34.23 ± 5.7 a	46.22 ± 7.25 cd	60.68 ± 4.49 bc
T2	39.76 ± 5.68 b	96.80 ± 14.47 a	11.18 ± 1.71 c	17.79 ± 2.40 b	26.46 ± 2.62 b	13.78 ± 3.33 b	153.59 ± 33.66 a	32.91 ± 8.89 a	96.04 ± 18.66 a	135.14 ± 22.06 a
T3	31.52 ± 13.45 b	68.02 ± 19.32 b	19.86 ± 3.92 b	35.11 ± 14.40 a	25.42 ± 8.49 b	25.34 ± 12.25 ª	92.16 ± 40.96 b	30.71 ± 8.89 a	89.65 ± 25.10 ab	102.56 ± 21.28 ab
T4	6.49 ± 0.56 c	5.33 ± 0.27 d	2.67 ± 0.13 d	1.79 ± 0.04 d	5.83 ± 0.81 c	2.65 ± 0.13 d	1.41 ± 0.01 c	2.24 ± 1.37 b	4.54 ± 0.38 e	7.19 ± 0.43 d
T5	22.94 ± 3.02 bc	29.52. ± 13.58 cd	10.28 ± 3.71 c	3.45 ± 0.91 cd	44.25 ± 18.34 a	7.91 ± 2.12 bcd	2.84 ± 0.91 c	0.00 ± 0.00 b	19.42 ± 6.76 de	29.60 ± 12.56 cd
T6	70.79 ± 19.89 a	58.57 ± 24.55 b	13.48 ± 4.60 bc	12.77 ± 1.98 bc	16.16 ± 3.31 bc	11.74 ± 1.45 bc	113.94 ± 50.34 ab	40.51 ± 16.92 a	67.54 ± 25.01 abc	136.62 ± 70.60 a

Antioxidant methodologies evaluated: Total phenols using the Folin–Ciocalteu method and where peak 1: caftaric acid; peak 2: 5-caffeolquinic acid; peak 3: caffeic acid; peak 4: coumaroylquinic acid; peak 5: caffeic acid derivative; peak 6: chlorogenic acid derivative; peak 7: chicoric acid isomer; peak 8: unidentified; peak 9: quercetin-3-glucuronide; peak 10: quercetin acetylhexoside derivative; Nps: Nanoparticles. Different letters indicate significant differences according to Tukey’s multiple range test (*p* < 0.05). Treatments: T0: control without inoculum; T1: *Candida guillermondi* with NPs; T2: *Candida guillermondi* without NPs; T3: *Rhodotorula mucilaginosa* with NPs; T4: *Rhodotorula mucilaginosa* without NPs; T5: consortium with NPs; T6: consortium without NPs.

## Data Availability

The data presented in this study are available upon request from the corresponding author.

## References

[1] (2020). Food and Agriculture Organization of the United Nations. FAOSTAT-Vegetables Production. https://www.fao.org/documents/card/en/c/cc3751en.

[2] Liu C., Chang C., Fei Y., Li F., Wang Q., Zhai G., Lei J. (2018). Cadmium accumulation in edible flowering cabbages in the Pearl River Delta, China: Critical soil factors and enrichment models. Environ. Pollut..

[3] Guo X., Luo J., Zhang R., Gao H., Peng L., Liang Y., Li T. (2022). Root cell wall remodeling mediates copper oxide nanoparticles phytotoxicity on lettuce (*Lactuca sativa* L.). Environ. Exp. Bot..

[4] Abd Rahim M., Hazrin-Chong N., Hazeera Harith H., Wan-Mohtar W., Sukor R. (2023). Roles of fermented plant-, dairy- and meat-based foods in the modulation of allergic responses. Food Sci. Hum. Wellness.

[5] Kim M.J., Moon Y., Tou J.C., Mou B., Waterland N.L. (2016). Nutritional value, bioactive compounds and health benefits of lettuce (*Lactuca sativa* L.). J. Food Compos. Anal..

[6] Al-Karaki G., Altuntas O. (2021). Growth, mineral content and antioxidant activity of romaine lettuce in relation to development stage in soilless system. Int. J. Agric. Sci. Technol..

[7] Al-Karaki G.N., Othman Y. (2023). Effect of foliar application of amino acid biostimulants on growth, macronutrient, total phenol contents and antioxidant activity of soilless grown lettuce cultivars. S. Afr. J. Bot..

[8] (2022). Statistics, Food and Agriculture Organization of the United Nations. FAOSTAT-Lettuce Production. http://www.fao.org/faostat/es/#data/QC.

[9] Yaseen A., Takacs-Hajos M. (2022). The effect of plant biostimulants on the macronutrient content and ion ratio of several lettuce (*Lactuca sativa* L.) cultivars grown in a plastic house. S. Afr. J. Bot..

[10] Santander C., Vidal G., Ruiz A., Vidal C., Cornejo P. (2022). Salinity Eustress Increases the Biosynthesis and Accumulation of Phenolic Compounds That Improve the Functional and Antioxidant Quality of Red Lettuce. Agronomy.

[11] Yang X., Lu M., Wang Y., Wang Y., Liu Z., Chen S. (2021). Response Mechanism of Plants to Drought Stress. Horticulturae.

[12] Sawatdee S., Prommuak C., Jarunglumlert T., Pavasant P., Flood A.E. (2021). Combined effects of cations in fertilizer solution on antioxidant content in red lettuce (*Lactuca sativa* L.). J. Sci. Food Agric..

[13] Materska M., Olszówka K., Chilczuk B., Stochmal A., Pecio T., Sienicka B., Piacente S., Pizza C., Masullo M. (2019). Polyphenolic profiles in lettuce (*Lactuca sativa* L.) after CaCl_2_ treatment and cold storage. Eur. Food Res. Technol..

[14] Mohd Yusof F.F., Yaacob J.S., Osman N., Ibrahim M.H., Wan-Mohtar W.A.A.Q.I., Berahim Z., Mohd Zain N.A. (2021). Shading. Effects on Leaf Gas Exchange, Leaf Pigments and Secondary Metabolites of *Polygonum minus* Huds., an Aromatic Medicinal Herb. Plants.

[15] Bhargava S., Sawant K. (2013). Drought stress adaptation: Metabolic adjustment and regulation of gene expression. Plant Breed..

[16] Govindasamy V., George P., Kumar M., Aher L., Raina S., Rane J., Annapurna K., Minhas P. (2020). Multi-trait PGP rhizobacterial endophytes alleviate drought stress in a senescent genotype of sorghum (*Sorghum bicolor* (L.) Moench). 3 Biotech.

[17] Jacoby R., Peukert M., Succurro A., Koprivova A., Kopriva S. (2017). The Role of Soil Microorganisms in Plant Mineral Nutrition—Current Knowledge and Future Directions. Front. Plant Sci..

[18] Silambarasan S., Logeswari P., Cornejo P., Abraham J., Valentine A. (2019). Simultaneous mitigation of aluminum, salinity and drought stress in *Lactuca sativa* growth via formulated plant growth promoting *Rhodotorula mucilaginosa* CAM4. Ecotoxicol. Environ. Saf..

[19] Silambarasan S., Logeswari P., Cornejo P., Kannan V. (2019). Evaluation of the production of exopolysaccharide by plant growth promoting yeast *Rhodotorula* sp. strain CAH2 under abiotic stress conditions. Int. J. Biol. Macromol..

[20] Alzandi A.A., Naguib D.M. (2022). Effect of yeast application on soil health and root metabolic status of corn seedlings under drought stress. Arch. Microbiol..

[21] Santander C., Ruiz A., García S., Aroca R., Cumming J., Cornejo P. (2020). Efficiency of two arbuscular mycorrhizal fungal inocula to improve saline stress tolerance in lettuce plants by changes of antioxidant defense mechanisms. J. Sci. Food Agric..

[22] Pérez R., Tapia Y., Antilén M., Casanova M., Vidal C., Silambarasan S., Cornejo P. (2021). Rhizosphere Management for Phytoremediation of Copper Mine Tailings. J. Soil Sci. Plant Nutr..

[23] Nasir Khan M., Mobin M., Abbas Z., AlMutairi K., Siddiqui Z. (2017). Role of nanomaterials in plants under challenging environments. Plant Physiol. Biochem..

[24] Feregrino-Perez A., Magaña-López E., Guzmán C., Esquivel K. (2018). A general overview of the benefits and possible negative effects of the nanotechnology in horticulture. Sci. Hortic..

[25] Rafique R., Zahra Z., Virk N., Shahid M., Pinelli E., Kallerhoff J., Park T.J., Arshad M. (2018). Data on rhizosphere pH, phosphorus uptake and wheat growth responses upon TiO_2_ nanoparticles application. Data Brief.

[26] Fatima F., Hashim A., Anees S. (2021). Efficacy of nanoparticles as nanofertilizer production: A review. Environ. Sci. Pollut..

[27] Gomez A., Narayan M., Zhao L., Jia X., Bernal R., Lopez-Moreno M., Peralta-Videa J. (2021). Effects of nano-enabled agricultural strategies on food quality: Current knowledge and future research needs. J. Hazard. Mater..

[28] Grillo R., Fraceto L., Amorim M., Scott-Fordsmand J., Schoonjans R., Chaudhry Q. (2021). Ecotoxicological and regulatory aspects of environmental sustainability of nanopesticides. J. Hazard. Mater..

[29] Fincheira P., Hoffmann N., Tortella G., Ruiz A., Cornejo P., Diez M.C., Seabra A.B., Benavides-Mendoza A., Rubilar O. (2023). Eco-Efficient Systems Based on Nanocarriers for the Controlled Release of Fertilizers and Pesticides: Toward Smart Agriculture. Nanomaterials.

[30] Lavicoli I., Leso V., Beezhold D., Shvedova A. (2017). Nanotechnology in agriculture: Opportunities, toxicological implications, and occupational risks. Toxicol. Appl. Pharmacol..

[31] Al-Mamun R., Hasan R., Ahommed S., Bacchu S., Ali R., Hossain Khan Z. (2021). Nanofertilizers towards sustainable agriculture and environment. Environ. Technol. Innov..

[32] Elanchezhian R., Kumar D., Ramesh K., Biswas A., Guhey A., Kumar Patra A. (2017). Morpho-physiological and biochemical response of maize (*Zea mays* L.) plants fertilized with nano-iron (Fe_3_O_4_) micronutrient. J. Plant Nutr..

[33] Lu K., Shen D., Liu X., Dong S., Jing X., Wu W., Tong Y., Gao S., Mao L. (2020). Uptake of iron oxide nanoparticles inhibits the photosynthesis of the wheat after foliar exposure. Chemosphere.

[34] Pariona N., Martinez A.I., Hdz-García H.M., Cruz L.A., Hernandez-Valdes A. (2017). Effects of hematite and ferrihydrite nanoparticles on germination and growth of maize seedlings. Saudi J. Biol. Sci..

[35] Du W., Yan J., Peng Q., Liang X., Mao H. (2019). Comparison study of zinc nanoparticles and zinc sulphate on wheat growth: From toxicity and zinc biofortification. Chemosphere.

[36] Hasan M., Rafique S., Zafar A., Loomba S., Khan R., Hassan S.G., Khan M.W., Zahra S., Zia M., Mustafa G. (2020). Physiological and anti-oxidative response of biologically and chemically synthesized iron oxide: *Zea mays* a case study. Heliyon.

[37] Pallavi C.M.M., Srivastava R., Arora S., Sharma A.K. (2016). Impact assessment of silver nanoparticles on plant growth and soil bacterial diversity. 3 Biotech.

[38] Nimsi K.A., Manjusha K., Kathiresan K., Arya H. (2023). Plant growth-promoting yeasts (PGPY), the latest entrant for use in sustainable agriculture: A review. J. Appl. Microbiol..

[39] Nassar A.H., El-Tarabily K.A., Sivasithamparam K. (2005). Promotion of plant growth by an auxin producing isolate of the yeast *Williopsis saturnus* endophytic in maize (*Zea mays* L.) roots. Biol. Fertil. Soils.

[40] Nutaratat P., Srisuk N., Arunrattiyakorn P., Limtong S. (2014). Plant growth-promoting traits of epiphytic and endophytic yeasts isolated from rice and sugar cane leaves in Thailand. Fungal Biol..

[41] Amprayn K.O., Rose M.T., Kecskés M., Pereg L., Nguyen H.T., Kennedy I.R. (2012). Plant growth promoting characteristics of soil yeast (*Candida tropicalis* HY) and its effectiveness for promoting rice growth. Appl. Soil Ecol..

[42] Hesham A.E.L., Mohamed H. (2011). Molecular genetic identification of yeast strains isolated from Egyptian soils for solubilization of inorganic phosphates and growth promotion of corn plants. J. Microbiol. Biotechnol..

[43] Bright J.P., Karunanadham K., Maheshwari H.S., Karuppiah E.A.A., Thankappan S., Nataraj R., Pandian D., Ameen F., Poczai P., Sayyed R.Z. (2022). Seed-Borne Probiotic Yeasts Foster Plant Growth and Elicit Health Protection in Black Gram (*Vigna mungo* L.). Sustainability.

[44] Lonhienne T., Mason M.G., Ragan M.A., Hugenholtz P., Schmidt S., Paungfoo-Lonhienne C. (2014). Yeasts as a biofertilizer alters plant growth and morphology. Crop Sci..

[45] Yang X., Wei S., Liu B., Guo D., Zheng B., Feng L., Liu Y., Tomás-Barberán F., Luo L., Huang D. (2018). A novel integrated non-targeted metabolomic analysis reveals significant metabolite variations between different lettuce (*Lactuca sativa*. L) varieties. Hortic. Res..

[46] Hameed M.K., Umar W., Razzaq A., Wei S., Niu Q., Huang D., Chang L. (2023). Quantification of total polyphenols, antioxidants, anthocyanins and secondary metabolites by UPLC VION IMS QTOF MS/MS analysis in green and red lettuce cultivars. Sci. Hortic..

[47] Llorach R., Martínez-Sánchez A., Tomás-Barberán F., Gil M., Ferreres F. (2008). Characterisation of polyphenols and antioxidant properties of five lettuce varieties and escarole. Food Chem..

[48] López A., García-Alonso J., Fenoll J., Hellín P., Flores P. (2014). Chemical composition and antioxidant capacity of lettuce: Comparative study of regular-sized (Romaine) and baby-sized (Little Gem and Mini Romaine) types. J. Food Compos. Anal..

[49] de Souza A.S.N., de Oliveira Schmidt H., Pagno C., Rodrigues E., da Silva M.A.S., Flôres S.H., de Oliveira Rios A. (2022). Influence of cultivar and season on carotenoids and phenolic compounds from red lettuce influence of cultivar and season on lettuce. Food Res. Int..

[50] Fincheira P., Espinoza J., Vera J., Berrios D., Nahuelcura J., Ruiz A., Quiroz A., Bustamante L., Cornejo P., Tortella G. (2023). The Impact of 2-Ketones Released from Solid Lipid Nanoparticles on Growth Modulation and Antioxidant System of *Lactuca sativa*. Plants.

[51] González F., Santander C., Ruiz A., Pérez R., Moreira J., Vidal G., Aroca R., Santos C., Cornejo P. (2023). Inoculation with *Actinobacteria* spp. Isolated from a Hyper-Arid Environment Enhances Tolerance to Salinity in Lettuce Plants (*Lactuca sativa* L.). Plants.

[52] Mahmoudi H., Huang J., Gruber M., Kaddour R., Lachaâl M., Ouerghi Z., Hannoufa A. (2010). The Impact of Genotype and Salinity on Physiological Function, Secondary Metabolite Accumulation, and Antioxidative Responses in Lettuce. J. Agric. Food Chem..

[53] Rebelo M.J., Rego R., Ferreira M., Oliveira M.C. (2013). Comparative study of the antioxidant capacity and polyphenol content of Douro wines by chemical and electrochemical methods. Food Chem..

[54] Cavalcanti V., Smail Aazza S., Bertolucci S., Pereira M., Cavalcanti P., Buttrós V., Oliveira e Silva A., Pasqual M., Dória J. (2020). Plant, pathogen and biocontrol agent interaction effects on bioactive compounds and antioxidant activity in garlic. Physiol. Mol. Plant Pathol..

[55] Zárate-Martínez W., González-Morales S., Ramírez-Godina F., Robledo-Olivo A., Juárez-Maldonado A. (2021). Effect of phenolic acids on the antioxidant system of tomato plants (*Solanum lycopersicum* Mill.). Agron. Mesoam..

[56] Avio L., Sbrana C., Giovannetti M., Frassinetti S. (2017). Arbuscular mycorrhizal fungi affect total phenolics content and antioxidant activity in leaves of oak leaf lettuce varieties. Sci. Hortic..

[57] Khan Z.S., Rizwan M., Hafeez M., Ali S., Adrees M., Qayyum M.F., Khalid S., Rehman M.Z.U., Sarwar M.A. (2020). Effects of silicon nanoparticles on growth and physiology of wheat in cadmium contaminated soil under different soil moisture levels. Environ. Sci. Pollut..

[58] García-López J.I., Niño-Medina G., Olivares-Sáenz E., Lira-Saldivar R.H., Barriga-Castro E.D., Vázquez-Alvarado R., Rodríguez-Salinas P.A., Zavala-García F. (2019). Foliar application of zinc oxide nanoparticles and zinc sulfate boosts the content of bioactive compounds in habanero peppers. Plants.

[59] García-Saucedo C., Field J., Otero-Gonzalez L., Sierra-Álvarez R. (2011). Low toxicity of HfO_2_, SiO_2_, Al_2_O_3_ and CeO_2_ nanoparticles to the yeast, *Saccharomyces cerevisiae*. J. Hazard. Mater..

[60] Otero-González L., García-Saucedo C., Field J., Sierra-Álvarez R. (2013). Toxicity of TiO_2_, ZrO_2_, Fe^0^, Fe_2_O_3_, and Mn_2_O_3_ nanoparticles to the yeast, *Saccharomyces cerevisiae*. Chemosphere.

[61] Ameen F., Alsamhary K., Alabdullatif J., ALNadhari S. (2021). A review on metal-based nanoparticles and their toxicity to beneficial soil bacteria and fungi. Ecotoxicol. Environ. Saf..

[62] Pérez R., Tapia Y., Antilén M., Ruiz A., Pimentel P., Santander C., Aponte H., González F., Cornejo P. (2023). Beneficial Interactive Effects Provided by an Arbuscular Mycorrhizal Fungi and Yeast on the Growth of *Oenothera picensis* Established on Cu Mine Tailings. Plants.

[63] Dasgan H.Y., Yilmaz D., Zikaria K., Ikiz B., Gruda N.S. (2023). Enhancing the Yield, Quality and Antioxidant Content of Lettuce through Innovative and Eco-Friendly Biofertilizer Practices in Hydroponics. Horticulturae.

[64] Harsela C.N. (2023). Growth and yields of bima brebes shallot variety planted using a floating hydroponics system. Eduvest-J. Univers. Stud..

[65] Kah M., Kookana R.S., Gogos A., Bucheli T.D. (2018). A critical evaluation of nanopesticides and nanofertilizers against their conventional analogues. Nat. Nanotechnol..

[66] Lopes M.J.S., Dias-Filho M.B., Gurgel E.S.C. (2021). Successful Plant Growth-Promoting Microbes: Inoculation Methods and Abiotic. Front. Sustain. Food Syst..

[67] Niksad N., Parastar H. (2021). Evaluation of the effect of organic pollutants exposure on the antioxidant activity, total phenolic and total flavonoid content of lettuce (*Lactuca sativa* L.) using UV–Vis spectrophotometry and chemometrics. Microchem. J..

[68] Favre G., González-Neves G., Piccardo D., Gómez-Alonso S., José Pérez-Navarro J., Hermosín-Gutiérrez I. (2018). New acylated flavonols identified in *Vitis vinifera* grapes and wines. Food Res. Int..

[69] Ou B., Chang T., Huang D., Prior R.L. (2013). Determination of Total Antioxidant Capacity by Oxygen Radical Absorbance Capacity (ORAC) Using Fluorescein as the Fluorescence Probe: First Action. J. AOAC Int..

